# Spanish validation of the pure procrastination scale: dimensional structure, internal consistency, temporal stability, gender invariance, and relationships with personality and satisfaction with life

**DOI:** 10.3389/fpsyg.2023.1268855

**Published:** 2024-01-17

**Authors:** Georgina Guilera, Maria Dolores López-Martínez, Maite Barrios, María Dolores Hidalgo, Piers Steel, Juana Gómez-Benito

**Affiliations:** ^1^Department of Social Psychology and Quantitative Psychology, Faculty of Psychology, University of Barcelona, Barcelona, Spain; ^2^Institute of Neurosciences, University of Barcelona, Barcelona, Spain; ^3^Department of Basic Psychology and Methodology, University of Murcia, Murcia, Spain; ^4^Haskayne School of Business, University of Calgary, Calgary, AB, Canada

**Keywords:** measures of procrastination, validity, reliability, measurement invariance, Spanish sample

## Abstract

The objective of the current study was to adapt and validate the pure procrastination scale (PPS) for the Spanish adult population. Procrastination can have numerous consequences in daily life, making it essential to have reliable and valid instruments for measuring procrastination. Thus, this study was conducted to address this need. The sample consisted of 596 adults aged 18–83 years (M = 35.25, SD = 13.75). In addition to the PPS, participants completed two procrastination measures, namely the irrational procrastination scale and the decisional procrastination questionnaire, alongside the Big Five inventory and the satisfaction with life scale. The results of the confirmatory factor analysis revealed a three-factor structure of the PPS. The examination of the reliability of scores in terms of internal consistency and temporal stability showed satisfactory results for the PPS scores. Moreover, gender invariance was observed at the scalar level. Finally, the PPS scores correlated with other measures of procrastination, personality traits, and satisfaction with life in the expected direction and magnitude. In conclusion, the Spanish PPS offers valid and reliable scores when administered to adult population.

## 1 Introduction

Among numerous interpretations of the procrastination phenomenon (e.g., Ferrari, 2001; Chun Chu and Choi, 2005; Simpson and Pychyl, 2009), a widely accepted definition refers to procrastination as the act of voluntarily delaying an intended course of action, despite anticipating that such a delay will result in negative consequences (Steel, 2007). Accordingly, procrastination is not just delay but an irrational delay, where we expect ourselves to be worse off for putting off. Any positive references to procrastination are relatively rare, with historical as well as philosophical interpretations consistent with it being knowingly risky or self-harmful. Klingsieck (2013) similarly arrives at an almost identical definition, that it is the voluntary delay of an intended and necessary and/or (personally) important activity, despite expecting potential negative consequences that outweigh the positive consequences of the delay. As can be seen, in both cases procrastination is an inherently irrational delay. In support of this, articles such as Chowdhury and Pychyl (2018) take explicit issue with variants such as active or purposeful procrastination, considering them oxymorons. Like many individual differences, procrastination can be studied at a trait level (i.e., a tendency to procrastinate across situations and time) or a state level (i.e., where we examine what conditions increase irrational delay). Though procrastination has sufficient stability to be considered a trait (Steel, 2007), along with a firm genetic component (Gustavson et al., 2014), it does fluctuate depending on the specific task (e.g., pleasant versus unpleasant) and an individual’s present condition (e.g., energetic versus tired). This goes by multiple names aside from state procrastination, including specific procrastination (Milgram et al., 1998), task-specific dilatory behavior (Hoppe et al., 2018), and momentary procrastination (Wieland et al., 2018). Consequently, people who do not consider themselves procrastinators (trait) may still procrastinate (state) on specific tasks, such as completing tax forms, writing wills, or dentist visits. Trait or state, procrastination is very common. One out of every five adults are thought to put off finishing their tasks, as suggested by various studies (Ferrari et al., 2007). The prevalence of procrastination is even higher among students, with 75% of college students self-identifying as procrastinators (Steel and Ferrari, 2013).

Regardless of the reasons that lead people to postpone their pre-planned duties and responsibilities (Steel, 2007; Burka and Yuen, 2008; Haghbin et al., 2012), procrastination behavior may increase stress and worry, lower mood, and negatively impact performance in school and at work (Sirois, 2007; Goroshit, 2018; Legood et al., 2018). Epidemiological research has also revealed a relationship between higher levels of trait procrastination with psychiatric symptomatology (i.e., anxiety and depression) and lower self-efficacy and satisfaction with life (van Eerde, 2003; Steel, 2007; Rozental et al., 2014; Duru and Balkis, 2017; Klein et al., 2017; Kim et al., 2020).

Adopting the Big Five personality framework (McCrae and Costa, 1989), numerous investigations have consistently reported a negative association between extraversion and emotional stability with trait procrastination (Chowdhury and Pychyl, 2018; Piotrowska, 2019; Zhou, 2019; Ocansey et al., 2020; Zanjani et al., 2020). Furthermore, several studies have extensively documented the relationship between conscientiousness and procrastination (Scher and Osterman, 2002; van Eerde, 2003; Lee et al., 2006; Steel, 2007; Steel and Klingsieck, 2016; Svartdal, 2017; Shaw and Zhang, 2021). In this regard, Steel and Klingsieck’s (2016) review found that conscientiousness and its facets (e.g., self-discipline) were strongly and negatively related to procrastination and appear to be central to procrastination, while other personality traits may not necessarily affect the degree of procrastination, but rather influence how it manifests in individuals. However, with a few exceptions (Rohrmann et al., 2016; Göncü Köse and Metin, 2018; Pearlman-Avnion and Zibenberg, 2018), the current body of research on the relationship between procrastination and personality traits is primarily constrained by the fact that most of the studies conducted to date have used samples of undergraduate students in academic settings. Consequently, it remains uncertain whether these findings generalize to other contexts and more diverse samples.

Insights into the sociodemographic characteristics of trait procrastination have been obtained from a recent meta-analysis study conducted by Lu et al. (2022). The findings of the study suggest that men tend to exhibit higher levels of procrastination than women for both general and academic procrastination. However, Lu et al. (2022) did not find any significant variations in procrastination tendencies based on other sociodemographic variables, including socioeconomic status, multiculturalism, nationality, family size, and educational background. Nevertheless, it is worth mentioning that the findings of this paper should be taken with some caution, since the studies included in this meta-analysis are mainly focused on Chinese population (93%), limiting the results generalizability of the results to other populations.

In these investigations, the use of adequate instruments to measure trait procrastination is imperative, which is our focus. Such tools ensure the accuracy and trustworthiness of the collected data, thereby enabling researchers to draw well-informed conclusions about procrastination. Different self-report measures have been developed to assess procrastination, as well as to test the underpinnings of different conceptualizations of procrastination. In this regard, the various available scales may differ primarily in their theoretical foundations, the specific assessment context for which they were designed, or their technical characteristics. Vangsness et al. (2022) assessed the psychometric properties of 10 procrastination measures and found that certain scales, e.g., irrational procrastination scale (IPS; Steel, 2010), pure procrastination scale (PPS; Steel, 2010), or Tuckman procrastination scale (TPS; Tuckman, 1991), displayed better psychometric properties than others, e.g., active procrastination scale (APS; Choi and Moran, 2009), adult inventory of procrastination (AIP; McCown and Johnson, 1989).

Despite the availability of measurement instruments in the Spanish linguistic and cultural context, i.e., the AIP, the decisional procrastination questionnaire (DPQ; Mann, 1982; Mann et al., 1997), the general procrastination scale (GPS; Lay, 1986), and the IPS, that have shown to provide effective and reliable measures (Díaz-Morales et al., 2006; Guilera et al., 2018), there exists a notable gap in the validation of the PPS for the Spanish-speaking population. The presence of robust measurement instruments in various languages is of paramount importance. Ensuring psychometrically-sound assessments across linguistic and cultural boundaries is not merely a matter of convenience but a fundamental requirement for meaningful and accurate research.

The PPS was developed by Steel (2010) based on three available instruments of procrastination (i.e., DPQ, GPS, and AIP). In his pioneering study, an exploratory factor analysis was performed with the items of the three scales. Those 12 items that loaded into the first factor were subsequently included in the PPS, resulting in a measure of “pure” procrastination. The PPS is currently available in 12 languages, i.e., Arabic (Besharat and Maserrat, 2019), Brazilian Portuguese (Rocha et al., 2021), English (Steel, 2010), Finnish (Svartdal et al., 2016), French (Rebetez et al., 2014), German (Svartdal et al., 2016), Italian (Svartdal et al., 2016), Japanese (Kaneko et al., 2022), Korean (Kim et al., 2020), Norwegian (Svartdal, 2017), Persian (Zamirinejad et al., 2022), Polish (Svartdal et al., 2016), and Swedish (Rozental et al., 2014). Furthermore, Díaz-Morales et al. (2006) translated into Spanish the items of the three instruments upon which the PPS is based, which were subsequently employed in the studies conducted by Codina et al. (2018, 2020) and Valenzuela et al. (2020). However, it is worth noting that none of these studies provided a comprehensive examination of the psychometric properties of the Spanish version of the PPS.

Numerous validation studies have shown satisfactory psychometric properties of the PPS, but one aspect that has generated some controversy refers to its dimensional structure. In the original study (Steel, 2010), although the dimensionality of the scale was not empirically tested, the author proposed a one-dimensional structure that derived from selecting those items from three different procrastination scales that loaded on the first factor in an exploratory factor analysis. Subsequently, alternative factorial structures have been proposed. With the Swedish version, Rozental et al. (2014) suggested the presence of two factors, both associated with the notion of voluntary delay. Rebetez et al. (2014) suggested another dimensional structure of the French version composed by two first-order factors (i.e., voluntary delay and observed delay), removing an item due to poor performance, with an additional second-order factor. In the study by Svartdal et al. (2016), the PPS was translated into several languages and the previously described factor structures were tested as well as a three-factor model in which items from the three original scales used to create the PPS were modeled as separate factors labeled decisional delay (i.e., delays in the decision-making phase), implemental delay (i.e., delays in actions) and lateness/timeliness (i.e., delays in meeting deadlines and punctuality). They found that the three-factor structure was the best fitting model across six European countries (Svartdal et al., 2016) and concluded that the PPS measures the three types of procrastination (i.e., decisional delay, implemental delay, and lateness/timeliness) even more adequately than the full scales from which the PPS is derived (Svartdal and Steel, 2017). Despite the multiplicity of plausible factorial structures, the structure characterized by three factors seems to be the most favorable (Svartdal et al., 2016; Svartdal and Steel, 2017; da Rocha, 2019; Rocha et al., 2021; Gagnon et al., 2022; Zamirinejad et al., 2022).

### 1.1 Aims of the study

The objective of the current study was to adapt and validate the PPS to the Spanish adult population through a rigorous process of adaptation and a comprehensive psychometric analysis. Specifically, our purposes were: (1) to study its dimensionality testing several competing models encountered in the literature; (2) to examine the reliability of scores in terms of internal consistency and temporal stability; (3) to test gender measurement invariance in order to explore the extent to which gender score comparisons are psychometrically justified; (4) to study the item performance, the adequacy of response categories, and the precision of the PPS in measuring different levels of procrastination by means of item response theory; and (5) to investigate the correlations between the PPS and other measures of procrastination, personality traits, and satisfaction with life. In this regard, it was hypothesized that the PPS scores would be: (a) strongly associated with the other measures of procrastination (Hypothesis 1) (Steel, 2010; Svartdal and Steel, 2017); (b) associated with measures of personality, strongly with conscientiousness, weakly with extraversion, agreeableness, and neuroticism, and negligibly with openness (Hypothesis 2) (Steel, 2007; Svartdal and Steel, 2017), and (c) negatively moderately correlated with satisfaction with life (Hypothesis 3) (Steel, 2010; Rebetez et al., 2014; Svartdal et al., 2016; Svartdal, 2017; Zamirinejad et al., 2022).

## 2 Methods

### 2.1 Participants

A total of 596 Spanish-speaking individuals from the general population (339 females and 257 males), aged 18–83 years (*M* = 35.25, *SD* = 13.75) participated in the study. Additional socio-demographic characteristics of the sample are shown in Table 1.

**Table 1:** Socio-demographic characteristics of the sample.

Variable	*n*	%	Variable	*n*	%
Gender			Living arrangement		
Men	257	43.1	Original family	183	30.7
Women	339	56.9	Own family	318	53.4
Employment			Friends	50	8.4
Wage earner	319	53.5	Alone	42	7.0
Self-employed	75	12.6	Other	3	0.5
Non-paid work	25	4.2	Educational level		
Unemployed	86	14.4	Primary education not completed	3	0.5
Retired	19	3.2	Primary education	58	9.7
Housewife	7	1.2	Secondary education	218	36.6
Disability	4	0.7	Higher education	284	47.7
Other	61	10.2	Other	33	5.5

### 2.2 Measures

The data collection protocol administered to the participants encompassed seven scales assessing happiness, life satisfaction, personality, grit, and procrastination. For the purposes of the current study, the three procrastination measures were included: the pure procrastination scale (PPS; Steel, 2010), the irrational procrastination scale (IPS; Steel, 2010), and the decisional procrastination questionnaire (DPQ; Mann et al., 1997). Additionally, the Big Five inventory (BFI; John et al., 1991) and the satisfaction with life scale (SWLS; Diener et al., 1985) were also included.

#### 2.2.1 Pure procrastination scale

The PPS (Steel, 2010) is formed by 12 items for evaluating procrastination conceptualized as a dysfunctional delay (e.g., “I am continually saying I’ll do it tomorrow”) that are to be answered on a 5-point Likert scale ranging from 1 (very seldom or not true of me) to 5 (very often true or true of me).

While Díaz-Morales et al. (2006) proposed a translation and adaptation into Spanish of the 12 items that later formed the PPS, two items were identified with divergent meanings in Spanish compared to the original English version. Consequently, the decision was made to reinitiate the process of translating and adapting these items. The development of the Spanish version of the PPS followed these steps. Firstly, two authors of this study (GG and MB) translated the 12 items from the original English version of the PPS into Spanish, taking into consideration the previously mentioned translation. Secondly, the Spanish version was translated back into English by an English-Spanish bilingual individual. Finally, any discrepancies between the original PPS and the back-translated version were thoroughly discussed among the authors, the bilingual individual, and the author of the English version (Steel, 2010). These discussions continued until a satisfactory solution was reached, ensuring that the original meaning of the items was preserved. It is noteworthy that all PPS items, except for the two problematic ones (i.e., item 9 and item 11), retained the exact wording as proposed in the study by Díaz-Morales et al. (2006), as it was deemed that their wording effectively maintained the intended meaning from the English version. The Spanish version of the PPS can be found in Table A1.

#### 2.2.2 Irrational procrastination scale

The IPS, developed by Steel (2010), consists of 9 items designed to evaluate irrational delays that lead to procrastination (e.g., “When I should be doing one thing, I will do another”). Participants respond to these items using a 5-point Likert scale, ranging from 1 = “very seldom or not true of me” to 5 = “very often true or true of me,” to assess the degree of procrastination. For the present study, the Spanish version of the IPS (Guilera et al., 2018) was utilized.

#### 2.2.3 Decisional procrastination questionnaire

Initially developed by Mann et al. (1997) the DPQ was used in its Spanish version by Díaz-Morales et al. (2006) in this study. This scale consists of 5 items, focusing on the tendency to put off decisions (e.g., “I do not make decisions unless I really have to”). Participants respond to these items using a 5-point Likert scale, ranging from 1 = “not true for me” to 5 = “true for me,” to indicate the degree to which each statement applies to them. Both the original and the Spanish version of the DPQ have demonstrated good psychometric properties when applied to the adult population.

#### 2.2.4 Big Five inventory

The BFI, developed by John et al. (1991) consists of 44 items presented in a 5-point Likert scale (ranging from 1 = “disagree strongly” to 5 = “agree strongly”). This inventory assesses the five major personality dimensions: Extraversion (e.g., “I see myself as someone who is full of energy”), agreeableness (e.g., “I see myself as someone who is generally trusting”), conscientiousness (e.g., “I see myself as someone who can be somewhat careless”), neuroticism (e.g., “I see myself as someone who is relaxed and handles stress well”), and openness (e.g., “I see myself as someone who has an active imagination”). In this study, the Spanish version of the BFI, as adapted by Benet-Martínez and John (1998), was utilized to measure these personality dimensions.

#### 2.2.5 Satisfaction with life scale

The SWLS, introduced by Diener et al. (1985), is a brief questionnaire comprising five items that assess life satisfaction (e.g., “The conditions of my life are excellent”). Participants rate their agreement with each statement on a scale from 1 = “strongly disagree” to 7 = “strongly agree.” In this study, the Spanish version of the SWLS was employed (Vázquez et al., 2013).

Supplementary Table S1 displays the descriptive statistics and internal consistency coefficients for the scores of the IPS, DPQ, BFI, and SWLS.

### 2.3 Procedure

For a self-report study involving adults from a community sample, obtaining approval from the Ethics Committee of the University of Barcelona was not necessary during the time of the study. Institutional review boards exempted the researchers from seeking approval for this type of research. Adult participants were recruited using a convenience sampling method, specifically the snowball approach. Prior to their participation, participants were fully informed about the research’s nature and objectives. They provided consent, understanding that their involvement was voluntary, and all data would be kept confidential throughout the study. Participants’ responses were collected using the Qualtrics platform. The sample recruitment process took place from October 2014 through November 2016.

### 2.4 Statistical analysis

The distribution of responses to PPS items was investigated by calculating the percentage of endorsement for each response category. To examine univariate normality, skewness and kurtosis values were computed for each item. Absolute values greater than 3 for skewness and 8 for kurtosis were considered to indicate extreme departures from normality (Nevitt and Hancock, 2000).

To examine the dimensional structure of the scale, the following competing models were fitted to the data by means of confirmatory factor analysis (CFA): (1) Model 1: one-factor model as originally proposed by Steel (2010); (2) Model 2: two-factor model proposed by Rozental et al. (2014) where items 4–8 load on a factor and items 1–3 and 9–12 load on another factor; (3) Model 3: two-factor model proposed by Rebetez et al. (2014) where items 1–8 are grouped into factor *voluntary delay* and items 9–11 into factor *observed delay* (i.e., note that item 12 is not included) and both factors load on a second-order factor; and (4) Model 4: three-factor model proposed by Svartdal et al. (2016) where items 1–3 load in a *decisional delay* factor, items 4–8 load in an *implemental delay* factor and items 9–12 in a *timeliness-lateness* factor. The models were tested using the weighted least square mean and variance adjusted (WLSMV) method, which is known to yield accurate parameter estimates when dealing with ordinal items, a small number of response categories, relatively small sample sizes, and non-multivariate normality (Li, 2016). Model fit was evaluated using several indices: Comparative fit index (CFI), Tucker-Lewis index (TLI), root mean square error approximation (RMSEA), and standardized root mean square residual (SRMR). To determine goodness of fit, the recommended guidelines were followed. Adequate fit was considered when CFI ≥0.95, TLI ≥0.95, RMSEA ≤0.06, and SRMR ≤0.08 (Hu and Bentler, 1999). An acceptable fit was considered when CFI ≥0.90, TLI ≥0.90, and RMSEA ≤0.08 (Bentler, 1990; Browne and Cudeck, 1993).

Internal consistency was assessed obtaining the Cronbach’s alpha coefficient (*α*) and McDonald’s omega (*ω*), considering that values around 0.90 are excellent, coefficients around 0.80 are very good, and values about 0.70 are adequate (Kline, 2016). To explore the test-retest reliability, the intraclass correlation coefficient (ICC) (i.e., single measures of absolute agreement) was computed relating scores from both administration times with values less than 0.50, between 0.50 and 0.75, between 0.75 and 0.90, and values greater than 0.90 being indicative of poor, moderate, good, and excellent reliability, respectively (Koo and Li, 2016).

Measurement invariance based on gender (i.e., men vs. women) was assessed using multigroup CFA in a sequential manner. The first step examined configural invariance to test if the factor structure of the PPS is equivalent across gender groups (i.e., if the model form of the PPS is equivalent for both men and women). This solution served as the baseline model for subsequent tests, where new model constraints were added in each step. The second step involved testing metric invariance, where factor loadings were constrained to be equal across gender groups. This step evaluated whether the items of the PPS contribute to the factors similarly for both genders. The third step examined scalar invariance by constraining item intercepts to be equal across groups. This step ensured that genders have the same baseline item average, allowing for valid comparisons of PPS scores between gender groups (Brown, 2015). To assess measurement invariance, Chen’s (2007) criteria were adopted. For metric invariance, the suggested thresholds were ΔCFI ≥ −0.010, ΔRMSEA ≥0.015, and ΔSRMR ≥0.030. For scalar invariance, the thresholds were ΔCFI ≥ −0.010, ΔRMSEA ≥0.015, and ΔSRMR ≥0.010. If the model showed a significant degradation in fit beyond these cut-offs, it would indicate measurement non-invariance at that specific step.

In order to examine item performance more in depth (e.g., item discrimination, the adequacy of items’ response categories), and to obtain measures of precision at different levels of the trait, the graded response model for polytomous items (GRM) (Samejima, 1969) was fitted for each of the three PPS factors (i.e., *decisional delay*, *implemental delay*, *timeliness*/*lateness*). A comparison between alternative models is displayed in Supplementary Table S2, showing that the GRM was the model that showed better fit to the data; thus, results are based on this model. Item fit was assessed by means of the generalized *χ*^2^ statistic (S-*χ*^2^), which indicates misfit when *p* values are lower than 0.05 (Orlando and Thissen, 2000; Kang and Chen, 2007), and by item infit and outfit indices, where values less than 0.5 or greater than 1.5 show potential misfit (De Ayala, 2009). Item discrimination and difficulty (i.e., threshold) parameters were estimated and interpreted following Baker (2001), where a value of 0.65 is used as the minimum threshold for an item to have acceptable discrimination, values between 1.35–1.69 indicate high item discrimination, and items with *a* > 1.70 are considered to have a very high discrimination level. To study item response categories suitability, the category response curves (CRC), which represent respondent’s probability of choosing one response category over another, were drawn for each PPS item. Additionally, the information function of both the items and the factors of the PPS were obtained.

Finally, the PPS scores were related with the IPS, DPQ, BFI and SWLS scores obtaining the Pearson correlation coefficient.

Analyses were conducted in R version 4.2.2 with the following packages: *lavaan* (Rosseel, 2012) for CFA and measurement invariance analysis, *semTools* (Jorgensen et al., 2022) for reliability coefficients, and *mirt* (Chalmers, 2012) for the IRT analysis.

## 3 Results

### 3.1 Item descriptives

Supplementary Table S3 presents the distribution of responses for each item in the PPS, along with descriptive statistics (mean, standard deviation, skewness, and kurtosis). Upon examining the distribution of item endorsement, it was observed that item 11 and item 12 had a floor effect, with approximately 80% of participants responding with either 1 = “very seldom or not true of me” or 2 = “seldom true of me.” However, the remaining items showed good coverage across the various response categories. Univariate normality analyses were conducted by calculating the skewness and kurtosis values for each item. The results indicated that skewness ranged from −0.26 to 1.59, and kurtosis ranged from −0.21 to 2.46, suggesting no strong deviation from normality.

### 3.2 Dimensional structure

The four competing models most commonly tested in the scientific literature were fitted to the data by means of CFA. Table 2 shows the goodness of fit indices of these models. The model that presented adequate fit was the three-factor model proposed by Svartdal et al. (2016), with items 1–3 loading in the *decisional delay* factor, items 4–8 loading in an *implemental delay* factor, and items 9–12 in a *timeliness-lateness* factor.

**Table 2:** Goodness of fit indices of the four factor structure models of the pure procrastination scale.

Models	*χ* ^2^	df	CFI	TLI	RMSEA (90% CI)	SRMR
1: One-factor model Steel (2010)	1167.13	54	0.875	0.847	0.186 (0.177–0.195)	0.093
2: Two-factor model Rozental et al. (2014)	779.03	53	0.918	0.898	0.152 (0.142–0.161)	0.085
3: Two-factor model Rozental et al. (2014)	309.84	42	0.969	0.959	0.104 (0.093–0.114)	0.064
4: Three-factor model Svartdal et al. (2016)	187.608	51	0.985	0.980	0.067 (0.057–0.078)	0.040

*χ*^2^, chi-square test of model fit; df, degrees of freedom; CFI, comparative fit index; TLI, Tucker-Lewis index; RMSEA, root mean square error of approximation; SRMR, standardized root mean square residual.

**Figure 1:**
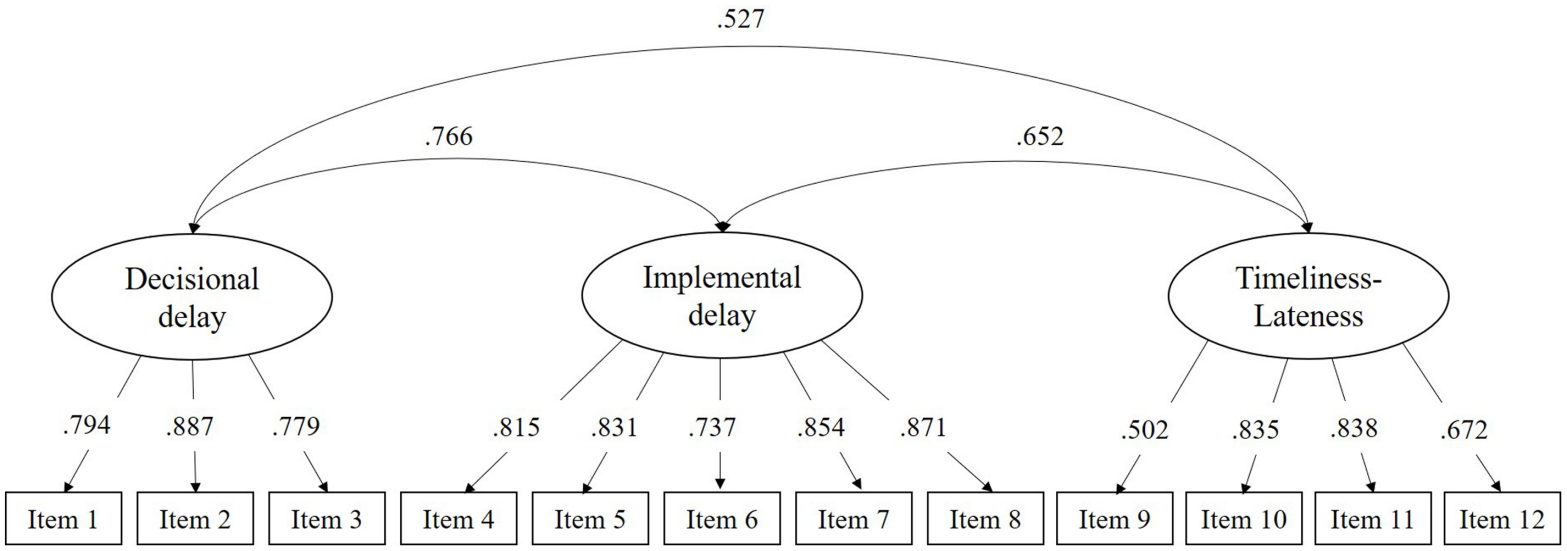
Path diagram of the three-factor model of the pure procrastination scale.

Figure 1 depicts the path diagram of the three-factor model in which lambdas and correlations between factors are shown. All factor loadings were high, statistically significant, and above the recommended value 0.40, and correlations between factors were moderately high.

### 3.3 Reliability

The internal consistency of the scores, measured by Cronbach’s alpha and McDonald’s omega coefficients, were, respectively, 0.82 and 0.82 for the *decisional delay* factor, 0.89 and 0.89 for the factor *implemental delay*, and 0.69 and 0.74 for *timeliness*/*lateness* factor. For the total score, Cronbach’s alpha and McDonald’s omega coefficients were, respectively, 0.90 and 0.92, indicating an excellent internal consistency of the PPS total score.

Temporal stability was tested for each subscale and total PPS scores. The ICCs values were 0.74 for *decisional delay* factor, 0.87 for the *implemental delay* factor, and 0.67 for *timeliness*/*lateness* factor. For the total score the ICC reached a value of 0.87. These results suggest from moderate (i.e., *decisional delay* and *timeliness*/*lateness* factors) to good (i.e., *implemental delay* factor and total score) test-retest reliability of the PPS scores.

### 3.4 Gender measurement invariance

Table 3 presents the results of the gender measurement invariance analyses, in which fit indices and differences in fit indices are shown for each step of the procedure. Accordingly, the PPS holds scalar invariance, allowing for comparison of scores between men and women. On average, men scored higher than women. Statistically significant differences were found in *decisional delay* and *implemental delay* factors and the total score (see Supplementary Table S4), but effect sizes were low.

**Table 3:** Fit indices for gender-based measurement invariance of the pure procrastination scale.

Model invariance	*χ* ^2^	df	CFI	RMSEA	SRMR	ΔCFI	ΔRMSEA	ΔSRMR
Configural	156.282	102	0.998	0.042	0.048	—	—	—
Metric	195.404	111	0.996	0.051	0.053	−0.002	0.009	0.005
Scalar	217.938	144	0.997	0.042	0.049	0.001	−0.009	−0.004

Configural: Baseline model; Metric: equal loadings; Scalar: equal loadings and intercepts. *χ*^2^, chi-square test of model fit; df, degrees of freedom; CFI, comparative fit index; RMSEA, root mean square error of approximation; SRMR, standardized root mean square residual; ΔCFI, change in CFI; ΔRMSEA, change in RMSEA; ΔSRMR: change in SRMR.

### 3.5 Item response theory analysis

IRT analyses were conducted separately for each of the three factors of the PPS. Table 4 depicts item fit statistics. According to the S-*χ*^2^ criteria, most of the items presented an adequate fit to the GRM, except items 2, 3 and 6, while according to infit and outfit values, item 1 was the one that showed an infit value out of the recommended range. Table 4 also shows the estimates of the item parameters, i.e., discrimination (*a*) and four difficulty parameters (*b*). According to Baker (2001), discrimination values for most of the items were very high (>1.70), while for item 12 was high (≥1.35) and for item 9 was acceptable (≥0.65). Results showed that *b* values of all items were ordered ascendingly (e.g., selecting a higher response category such as 5 = “very often true or true of me” requires higher levels of the latent trait), and that items cover a wide range of the latent trait.

**Table 4:** Item statistics for the graded response model (GRM) across the items of the pure procrastination scale.

Items	*a*	*b*1	*b*2	*b*3	*b*4	S-*χ*^2^	df	Infit	Outfit
**Decisional**									
PPS 1	3.39	−0.90	0.18	1.37	2.32	8.39	5	1.61	0.56
PPS 2	2.84	−1.06	0.20	1.22	2.51	12.66^*^	5	0.73	0.71
PPS 3	2.09	−1.36	−0.00	0.80	2.28	14.92^*^	7	0.80	0.78
**Implemental**									
PPS 4	2.52	−1.33	−0.13	0.63	1.79	34.651	26	0.84	0.85
PPS 5	2.72	−1.18	0.03	0.90	1.92	27.311	23	0.80	0.83
PPS 6	2.00	−1.65	−0.26	0.93	2.45	56.379^**^	25	0.95	0.94
PPS 7	2.96	−1.12	−0.04	0.83	1.91	18.387	23	0.76	0.79
PPS 8	3.57	−1.30	−0.12	0.73	1.82	12.119	20	0.69	0.73
**Timeliness/lateness**									
PPS 9	0.69	−4.83	−2.10	0.14	2.77	27.384	17	0.97	0.95
PPS 10	3.33	−0.59	0.91	1.81	2.37	13.039	14	0.69	0.62
PPS 11	3.23	−0.14	1.24	1.85	2.53	17.136	15	0.83	0.65
PPS 12	1.57	0.34	1.62	2.64	3.43	31.635	20	0.95	0.81

PPS, pure procrastination scale; *a*, item discrimination; *b*, item difficulty/threshold; S-*χ*^2^, generalized chi-squared statistic; df, degrees of freedom. ^*^p < 0.05 and ^**^p < 0.01.

**Figure 2:**
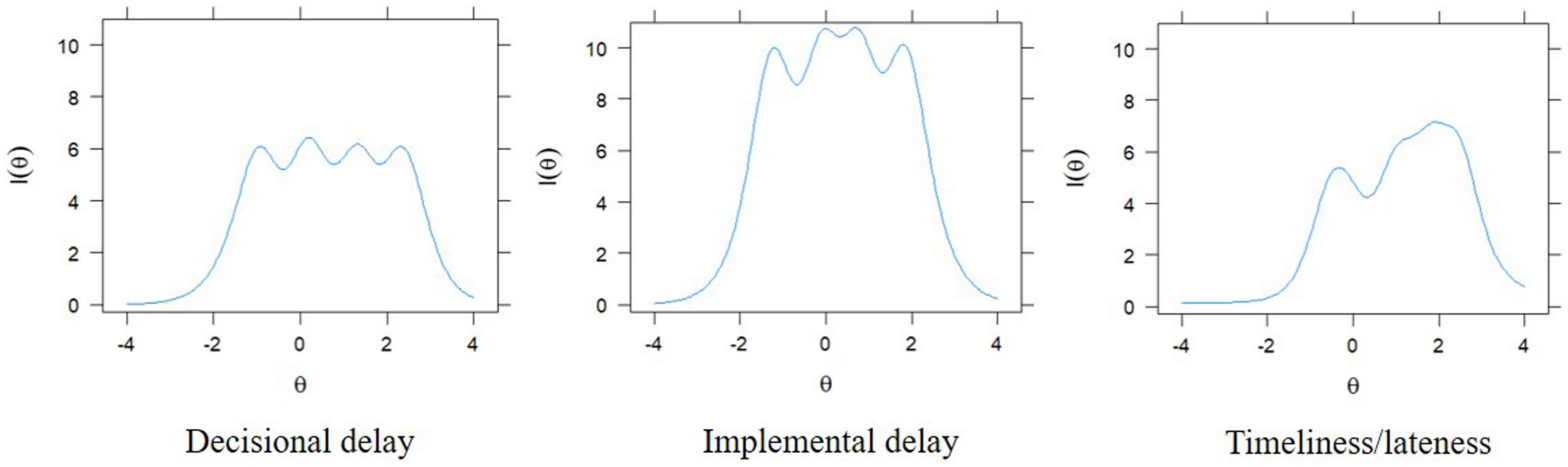
Information functions for the three factors of the pure procrastination scale.

By examining Supplementary Figure S1, it becomes evident that the response curves for most PPS items exhibited satisfactory separation and did not overlap, indicating that the response categories performed well. However, in item 12, the response curves for the categories “4 = often true of me” and “5 = very often true or true of me” showed some overlap, suggesting that participants experienced slight difficulty distinguishing between these options. Additionally, Supplementary Figure S2 displays the item information functions of the PPS items. Regarding the test information functions of the three dimensions (see Figure 2), results showed that the PPS was more precise at levels of the latent trait from −1 theta values to 2 for *decisional delay*, from −2 to 2 for *implemental delay*, and from 1 to 2 for *timeliness*/*lateness*.

### 3.6 Correlations between the PPS and other measures of procrastination, personality traits and satisfaction with life

To evaluate the external validity of the Spanish version of the PPS, Pearson correlations were computed between the scores of the PPS (the total score and the subscale’s scores) with the IPS, the DPQ, the BFI traits and the SWLS (see Table 5). The PPS total score strongly correlated with the IPS and the DPQ (0.86 and 0.81, respectively), supporting Hypothesis 1. On the other hand, the correlation between the PPS total score and the Conscientiousness factor of the BFI was −0.69, and with the BFI factors extraversion, agreeableness, and neuroticism were all low and almost null with the openness factor, supporting Hypothesis 2. Finally, the PPS total score and the SWLS were negatively associated with moderate strength (*r* = −0.35), supporting Hypothesis 3. Table 5 shows additional correlations of all these measures with the three factors of the PPS separately.

**Table 5:** Correlations between the pure procrastination scale (total and subscales) and other measures of procrastination, personality traits and satisfaction with life.

Measures	Correlations with PPS scores			
	Decisional	Implemental	Timeliness/lateness	Total
IPS	0.67^**^	0.83^**^	0.61^**^	0.86^**^
DPQ	0.86^**^	0.70^**^	0.51^**^	0.81^**^
BFI-extraversion	−0.35^**^	−0.21^**^	−0.17^**^	−0.28^**^
BFI-agreeableness	−0.25^**^	−0.22^**^	−0.19^**^	−0.26^**^
BFI-conscientiousness	−0.50^**^	−0.68^**^	−0.50^**^	−0.69^**^
BFI-neuroticism	0.26^**^	0.20^**^	0.23^**^	0.27^**^
BFI-openness	−0.16^**^	0.00	−0.03	−0.06
SWLS	−0.32^**^	−0.29^**^	−0.27^**^	−0.35^**^

PPS, pure procrastination scale; IPS, irrational procrastination scale; DPQ, decisional procrastination questionnaire; BFI, Big Five inventory; SWLS, satisfaction with life scale. ^*^p < 0.05 and ^**^p < 0.01.

## 4 Discussion

The main objective of the present study was to adapt and validate the PPS into the Spanish cultural context. Results suggest that the PPS performs adequately in adult population and may be a suitable instrument for assessing procrastination in epidemiological and/or cross-cultural studies.

Regarding the internal structure of the PPS, a three-factor structure was found, representing *decisional delay* (items 1 to 3), *implemental delay* (items 4 to 8), and *timeless*/*lateness* (items 9 to 12). These results are in line of a vast number of studies in which the same dimensional structure is proposed (Svartdal et al., 2016; Svartdal and Steel, 2017; Rocha et al., 2021; Gagnon et al., 2022; Kaneko et al., 2022; Zamirinejad et al., 2022).

Results of the present study also demonstrate adequate internal consistency values of the PPS scores. Thus, when scores in each factor were considered, internal consistency was adequate for the *decisional delay* and *implemental delay* subscales, while was limit for the *timeliness*/*lateness*. In general, except for the *timeliness*/*lateness* factor, these results are in line with those found in other countries. In the study conducted by Svartdal et al. (2016), where PPS was applied in six different countries (i.e., Finland, Germany, Italy, Norway, Poland and Sweden), Cronbach’s alpha range was 0.75 to 0.84 for *decisional delay*, 0.88 to 0.93 for *implemental delay*, and 0.71 to 0.80 for *timeless*/*lateness*. Results obtained in other cross-cultural adaptations showed the same trend, with lower internal consistency values for the *decisional delay* and *timeless*/*lateness* dimensions. Accordingly, in the Persian adaptation Cronbach’s alpha coefficients were 0.83 (*decisional delay*), 0.90 (*implemental delay*) and 0.85 (*timeless*/*lateness*) (Zamirinejad et al., 2022), in Brazilian adaptation were 0.80, 0.90 and 0.80 (Rocha et al., 2021), and in the global worldwide study by Svartdal and Steel (2017), in which participants were from countries around the world mostly English-speaking, Cronbach’s alpha coefficients were 0.83 (*decisional delay*), 0.87 (*implemental delay*) and 0.85 (*timeless*/*lateness*). As noted by Svartdal et al. (2016), *timeless*/*lateness* dimension seemed to be particularly sensitive to cultural and subgroup differences. Thus, in our study the low internal consistency value for the *timeless*/*lateness* dimension may be due to the lower variability of the scores on this factor and a lower average value with respect to the other factors in the Spanish culture. Moreover, internal consistency of the Spanish PPS total score is satisfactory, being similar to those reported by Svartdal et al. (2016) (i.e., range over countries: 0.89–0.93), by Svartdal and Steel (2017) (i.e., 0.92) in the global worldwide study, by Zamirinejad et al. (2022) (i.e., 0.94) in the Persian version, by Rocha et al. (2021) (i.e., 0.91) in the Brazilian Portuguese version, and in the Japanese version by Kaneko et al. (2022) (i.e., 0.92).

The assessment of temporal stability suggests from moderate (i.e., *decisional delay* and *timeliness*/*lateness* factors) to good (i.e., *implemental delay* factor) test-retest reliability coefficients. Results obtained in other adaptations have been higher as in the case of the Persian version (Zamirinejad et al., 2022) where test-retest reliabilities were 0.81 (*decisional delay*), 0.80 (*implemental delay*), 0.79 (*timeliness*/*lateness*) or lower as the Japanese version (Kaneko et al., 2022), where coefficients were 0.72, 0.62, and 0.54, respectively. For the PPS total score test-retest reliability obtained in this study was similar to that shown in the French version (Rebetez et al., 2014) (i.e., 0.87), the Persian version (Zamirinejad et al., 2022) (i.e., 0.88) and higher than that obtained in the Japanese version (Kaneko et al., 2022) (i.e., 0.68).

Moreover, measurement invariance by gender was examined for the Spanish version of the PPS. The presence of scalar invariance by gender indicates that PPS scores can be adequately compared between men and women. Consequently, researchers and users of the PPS can confidently compare mean scores between men and women, as the procrastination construct, measured by the PPS, holds the same meaning for both groups. The results of this comparison suggest differences between men and women in the PPS total score with a small effect size, being the average score higher for men. On the other hand, differences in the same direction and magnitude were also found in the scores of the *decisional delay* and *implemental delay* factors. Regarding the invariance of the measure by gender, our results are partially in line with those found by Svartdal and Steel (2017). They found full scalar invariance for the *implemental delay* factor, and configural and metric invariance for *decisional* and *lateness* factors. With respect to the differences between means by gender, the results of this study are similar to those reported in other studies, such as those found by Steel and Ferrari (2013) and Svartdal (2017), with men tending to have higher PPS scores that women, and by Svartdal et al. (2016) with men having higher scores than women only for *implemental delay* factor, over all countries analyzed, except for Norway where men had higher scores on all three factors compared to women.

The results of IRT analyses indicated that the PPS items exhibited adequate properties in terms of discrimination, difficulty, and the overall information they collectively provided. The majority of items demonstrated high discrimination parameter values, implying that they effectively differentiate between various levels of procrastination. Even though item 9 exceeded the minimum threshold for an item to have acceptable discrimination, it showed the lowest value compared to the remaining items, as found in previous studies (Svartdal and Steel, 2017). Difficulty parameters suggested that the PPS covers different levels of procrastination, since they covered a wide range of the ability range. PPS items were more informative at medium-high levels of the different dimensions of procrastination. Item 9 was less informative and did not achieve the minimum required. As noted by Steel (2010), p. 930, item 9 (“I find myself running out of time”) may be measuring busyness and not procrastination *per se*. All together these results suggest that the PPS would be suitable to differentiate between persons who procrastinate to some degree and those who do not in terms of decisional delay, implemental delay, and timeless/lateness.

Correlations between PPS scores and other measures of procrastination, personality, and life satisfaction were in the expected direction and magnitude, suggesting evidence of validity. Additionally, correlations between PPS and IPS scores were consistent with previous research (Rozental et al., 2014; Svartdal et al., 2016; Svartdal, 2017; Svartdal and Steel, 2017; Kim et al., 2020; Rocha et al., 2021). Svartdal et al. (2016), for example, found high correlation coefficients between PPS total score and IPS scores across different countries, ranging from 0.79 to 0.89 (with a coefficient of 0.86 in our study). Similarly, they found correlations between IPS and the *implemental delay* factor in the range of 0.79 to 0.87 (i.e., 0.83 in our study), correlations with the *decisional delay* factor in the range of 0.61 to 0.69 (i.e., 0.67 in our study), and correlations with the *timeless*/*lateness* factor in the range of 0.50 to 0.71 (i.e., 0.61 in our study). This pattern of high correlations of the IPS with PPS and *implemental delay* factor and more moderate correlations with the two other factors are in line with the expected, providing evidence of convergent validity. Furthermore, there was a significant negative relationship between the scores of the PPS and the SWLS, suggesting that they measure contrasting constructs. This finding is consistent with prior research (Rebetez et al., 2014; Svartdal, 2017; Svartdal and Steel, 2017; Kaneko et al., 2022). Finally, the correlations observed with the Big Five personality traits align with those reported in other studies (van Eerde, 2003; Steel, 2007; Rebetez et al., 2014), which indicate that procrastination is strongly inversely related to conscientiousness.

Altogether, these results provide evidence of the validity of the PPS in measuring trait procrastination and can be used in future research to better understand the complex relationships between procrastination, personality, and life satisfaction.

### 4.1 Limitations

It is important to acknowledge certain limitations of this study. First, the sample was recruited through convenience sampling, in which participants were self-selected. This sampling method may limit the generalizability of the findings to the broader Spanish population. Second, the sample composition may not fully represent the diversity of the Spanish population in terms of educational attainment. Specifically, individuals with primary education or lower were underrepresented, while those with secondary education were overrepresented in comparison to the characteristics of the Spanish population. Third, data collection concluded at the end of 2016, so the data is partially recent.

### 4.2 Research implications

The present study represents a significant advancement in the study of procrastination within the Spanish cultural context. We provide a new measurement tool for assessing trait procrastination in Spanish-speaking settings, which is now available to the scientific community. Furthermore, this study offers new insights into how procrastination can be measured, specifically through decisional delay, implemental delay, and timeless/lateness.

To build upon these findings, further research is needed to explore the applicability of the PPS as a measure of procrastination in Spanish-speaking countries. Replication of our results in representative samples is necessary to establish the generalizability of our findings. Additionally, future studies should investigate measurement invariance across different cultural and linguistic versions of the PPS.

## 5 Conclusion

The current study offers evidence for the validity of the three-factor structure of the Spanish PPS, as well as its internal consistency and temporal stability. Additionally, we found that the instrument exhibits measurement invariance across gender, with good item performance and appropriate response categories. The PPS effectively measures the spectrum of procrastination and shows significant correlations with other measures of trait procrastination and related constructs. Overall, our findings suggest that the Spanish PPS is a suitable instrument for use in large studies to determine the prevalence and severity of self-reported procrastination. The ease of administration and precise measurement of the three procrastination traits (i.e., decisional delay, implemental delay, and timeless/lateness) make it a valuable tool for researchers interested in investigating this phenomenon.

## Data availability statement

The raw data supporting the conclusions of this article will be made available by the authors, without undue reservation.

## Ethics statement

Ethical review and approval was not required for the study on human participants in accordance with the local legislation and institutional requirements. The studies were conducted in accordance with the local legislation and institutional requirements. The participants provided their written informed consent to participate in this study.

## Author contributions

GG: Formal analysis, Methodology, Software, Visualization, Writing – original draft, Conceptualization, Data curation, Funding acquisition, Investigation, Project administration, Resources, Supervision, Validation. ML-M: Writing – original draft, Formal analysis, Methodology, Software, Visualization. MB: Conceptualization, Data curation, Investigation, Validation, Writing – original draft. MH: Conceptualization, Methodology, Software, Writing – original draft. PS: Methodology, Writing – review & editing. JG-B: Funding acquisition, Investigation, Project administration, Resources, Writing – review & editing.

## Appendix

**Table A1:** Spanish version of the pure procrastination scale.

Instrucciones: Lea con atención las siguientes afirmaciones y piense si describen o no lo que le sucede. No existen dos afirmaciones iguales, por lo tanto, le pedimos que considere cada una cuidadosamente antes de responder. Responda lo más honestamente posible, de acuerdo con la siguiente escala: 1 = No me describe en absoluto 2 = No es usual en mí 3 = A veces sí, a veces no 4 = Es usual en mí 5 = Me describe totalmente
1. Retraso tanto mis decisiones que cuando me decido ya es demasiado tarde *[I delay making decisions until it’s too late]*
2. Incluso después de haber tomado una decisión, me demoro en llevarla a cabo *[Even after I make a decision I delay acting upon it]*
3. Pierdo bastante tiempo en detalles sin importancia antes de tomar la decisión final *[I waste a lot of time on trivial matters before getting to the final decisions]*
4. Cuando estoy haciendo un trabajo que debo presentar, con frecuencia pierdo tiempo haciendo otras cosas *[In preparation for some deadlines, I often waste time by doing other things]*
5. Tardo varios días en realizar trabajos, incluso los que sólo requieren sentarse y hacerlos *[Even jobs that require little else except sitting down and doing them, I find that they seldom get done for days]*
6. Frecuentemente me doy cuenta que estoy haciendo tareas que me había propuesto hacer con anterioridad *[I often find myself performing tasks that I had intended to do days before]*
7. Estoy continuamente diciendo: “lo haré mañana” *[I am continually saying “I’ll do it tomorrow”]*
8. Generalmente me demoro en comenzar el trabajo que tengo que hacer *[I generally delay before starting on work I have to do]*
9. Siento que siempre me falta tiempo *[I find myself running out of time]*
10. No tengo las cosas hechas a tiempo *[I do not get things done on time]*
11. Soy una persona poco cumplidora con los plazos *[I am not very good at meeting deadlines]*
12. Retrasar las cosas hasta el último minuto me ha costado dinero *[Putting things off till the last minute has cost me money in the past]*
