# Supplementary material for: Spanish validation of the pure procrastination scale: dimensional structure, internal consistency, temporal stability, gender invariance, and relationships with personality and satisfaction with life
Source: Front Psychol. 2024 Jan 17;14:1268855. doi: 10.3389/fpsyg.2023.1268855 (PMC10828008; doi:10.3389/fpsyg.2023.1268855)
Supplement: Supplementary file 1 [file Table_1.pdf]

Supplementary Material

Supplementary Table 1

Table S1 Descriptive statistics and reliability coefficients of the Irrational Procrastination Scale, the Decisional Procrastination Questionnaire, the Big Five Inventory, and the Satisfaction With Life Scale

Measures	Mean	*SD*	Range	Alpha/Omega
IPS	23.28	6.60	9-45	.90/.90
DPQ	11.87	4.24	5-25	.90/.90
BFI-Extraversion	27.05	6.41	8-40	.84/.87
BFI-Agreeableness	34.32	5.18	17-45	.70/.70
BFI-Conscientiousness	32.93	6.23	10-45	.82/.84
BFI- Neuroticism	22.94	6.34	9-40	.83/.84
BFI-Openness	38.36	6.45	16-50	.81/.85
SWLS	23.64	6.09	6-35	.85/.84

Note: IPS: Irrational Procrastination Scale; DPQ: Decisional Procrastination Questionnaire; BFI: Big Five Inventory; SWLS: Satisfaction With Life Scale
