# Supplementary material for: Spanish validation of the pure procrastination scale: dimensional structure, internal consistency, temporal stability, gender invariance, and relationships with personality and satisfaction with life
Source: Front Psychol. 2024 Jan 17;14:1268855. doi: 10.3389/fpsyg.2023.1268855 (PMC10828008; doi:10.3389/fpsyg.2023.1268855)
Supplement: Supplementary file 2 [file Table_2.pdf]

Supplementary Material

Supplementary Table 2

Table S2 Comparative fit of the Partial Credit Model, the Generalized Partial Credit Model, the Graded Response Model, and the Rating Scale Model for each subscale

Subscale	AIC	BIC	Best-fitting model
Decisional delay			
PCM	4368.45	4425.52	GRM
GPCM	4354.35	4420.20	
GRM	4330.85	4396.70	
RSM	4396.23	4426.96	
Implemental delay			
PCM	7211.40	7303.59	GRM
GPCM	7179.49	7289.25	
GRM	7126.94	7236.69	
RSM	7227.81	7267.32	
Timeliness/lateness			
PCM	5429.16	5503.80	GRM
GPCM	5284.21	5372.02	
GRM	5220.35	5308.15	
RSM	5490.07	5525.19	

Note: PCM: partial credit model; GPCM: generalized partial credit model; GRM: graded response model; RSM: rating scale model; AIC: Akaike information criterion; BIC: Bayesian information criterion
