# Supplementary material for: Spanish validation of the pure procrastination scale: dimensional structure, internal consistency, temporal stability, gender invariance, and relationships with personality and satisfaction with life
Source: Front Psychol. 2024 Jan 17;14:1268855. doi: 10.3389/fpsyg.2023.1268855 (PMC10828008; doi:10.3389/fpsyg.2023.1268855)
Supplement: Supplementary file 3 [file Table_3.pdf]

Supplementary Material

Supplementary Table 3

Table S3 Item endorsements in each response category and corresponding skewness and kurtosis values

	Response category *n* (%)							
Item	1	2	3	4	5	Mean (*SD*)	Skewness	Kurtosis
PPS 1	123 (20.6)	216 (36.2)	194 (32.6)	49 (8.2)	14 (2.3)	2.35 (.97)	.39	-0.21
PPS 2	106 (17.8)	235 (39.4)	170 (28.5)	73 (12.2)	12 (2.0)	2.41 (.98)	.39	-0.39
PPS 3	86 (14.4)	215 (36.1)	139 (23.3)	130 (21.8)	26 (4.4)	2.66 (1.10)	.26	-0.84
PPS 4	80 (13.4)	195 (32.7)	143 (24.0)	135 (22.7)	43 (7.2)	2.78 (1.15)	.20	-0.89
PPS 5	94 (15.8)	215 (36.1)	156 (26.2)	98 (16.4)	33 (5.5)	2.60 (1.10)	.38	-0.60
PPS 6	63 (10.6)	187 (31.4)	206 (34.6)	120 (20.1)	20 (3.4)	2.74 (1.00)	.10	-0.59
PPS 7	98 (16.4)	193 (32.4)	166 (27.9)	109 (18.3)	30 (5.0)	2.63 (1.11)	.26	-0.73
PPS 8	71 (11.9)	202 (33.9)	172 (28.9)	120 (20.1)	31 (5.2)	2.73 (1.07)	.23	-0.69
PPS 9	25 (4.2)	99 (16.6)	186 (31.2)	197 (33.1)	89 (14.9)	3.38 (1.06)	-.26	-0.58
PPS 10	178 (29.9)	290 (48.7)	95 (15.9)	23 (3.9)	10 (1.7)	1.99 (0.87)	.97	1.22
PPS 11	265 (44.5)	244 (40.9)	55 (9.2)	25 (4.2)	7 (1.2)	1.77 (0.87)	1.28	1.70
PPS 12	351 (58.9)	163 (27.3)	58 (9.7)	16 (2.7)	8 (1.3)	1.60 (0.87)	1.59	2.46

Note: 1 = “very seldom or not true of me”; 2 = “seldom true of me”; 3 = “sometimes true of me”; 4 = “often true of me”; 5 = “very often true or true of me”
