# Supplementary material for: Spanish validation of the pure procrastination scale: dimensional structure, internal consistency, temporal stability, gender invariance, and relationships with personality and satisfaction with life
Source: Front Psychol. 2024 Jan 17;14:1268855. doi: 10.3389/fpsyg.2023.1268855 (PMC10828008; doi:10.3389/fpsyg.2023.1268855)
Supplement: Supplementary file 4 [file Table_4.pdf]

Supplementary Material

Supplementary Table 4

Table S4 Comparison between men and women

Factor	Gender	*n*	Mean (*SD*)	*t*(df)	*p* value	Cohen's *d*
Decisional delay	Men	257	7.72 (2.69)	2.41(594)	.016	0.20
	Women	339	7.20 (2.56)			
Implemental delay	Men	257	14.01 (4.35)	2.52(594)	.012	0.21
	Women	339	13.07 (4.64)			
Timeliness/lateness	Men	257	8.93 (2.87)	1.59(594)	.113	0.13
	Women	339	8.59 (2.46)			
Total score	Men	257	30.67 (8.27)	2.64(594)	.008	0.22
	Women	339	28.86 (8.30)			
