# Supplementary material for: Spanish validation of the pure procrastination scale: dimensional structure, internal consistency, temporal stability, gender invariance, and relationships with personality and satisfaction with life
Source: Front Psychol. 2024 Jan 17;14:1268855. doi: 10.3389/fpsyg.2023.1268855 (PMC10828008; doi:10.3389/fpsyg.2023.1268855)

Supplementary Material

Supplementary Figure 1

Figure S1 Category Response Curves (CRC) of the items

a) *Decisional delay* factor

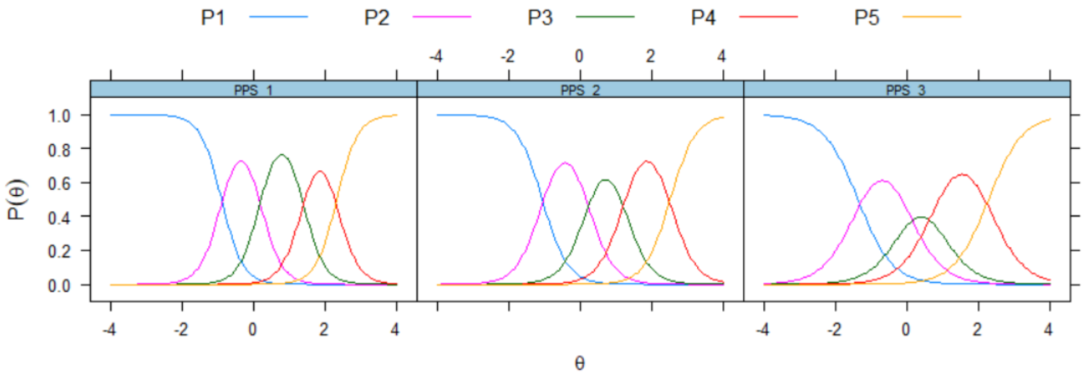

b) *Implemental delay* factor

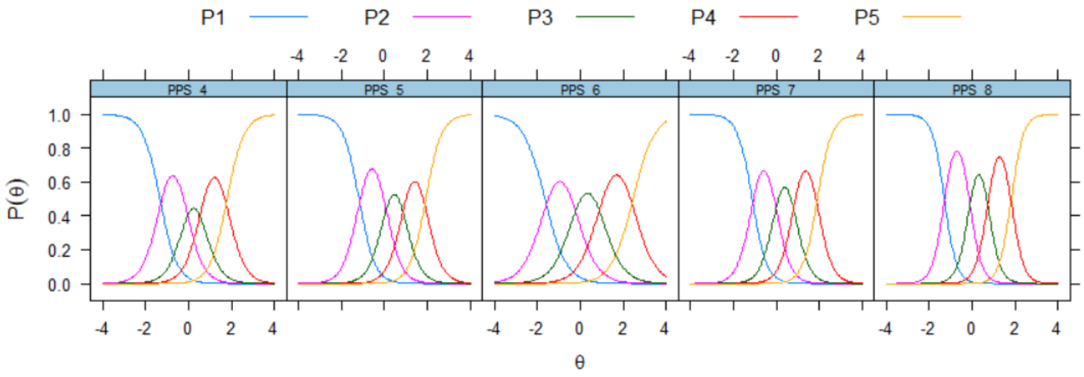

c) *Timeliness/lateness* factor

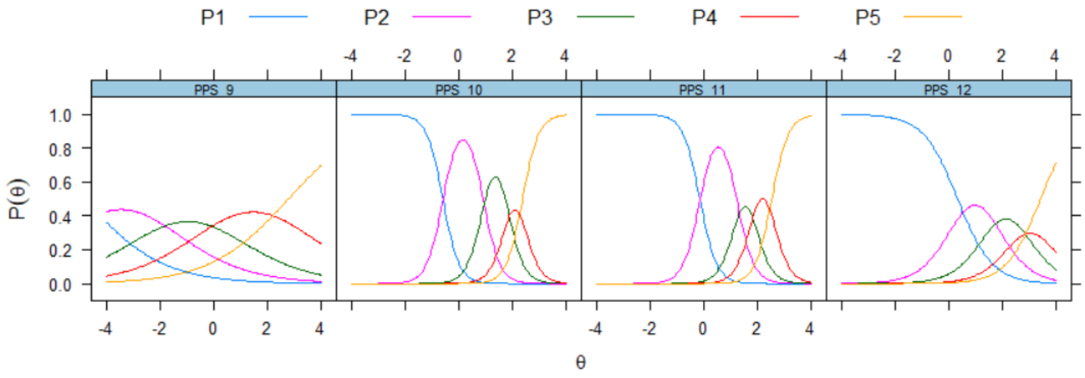

Supplement: Supplementary file 5 [file Image_1.pdf]
